# Pharmacokinetic Parameters and Estimated Milk Withdrawal Intervals for Domestic Goats (*Capra Aegagrus Hircus*) After Administration of Single and Multiple Intravenous and Subcutaneous Doses of Flunixin Meglumine

**DOI:** 10.3389/fvets.2020.00213

**Published:** 2020-05-19

**Authors:** Joe S. Smith, Tara L. Marmulak, John A. Angelos, Zhoumeng Lin, Joan D. Rowe, Jan L. Carlson, Weilin L. Shelver, Elizabeth A. Lee, Lisa A. Tell

**Affiliations:** ^1^School of Veterinary Medicine, William R. Pritchard Veterinary Medical Teaching Hospital (VMTH), University of California, Davis, Davis, CA, United States; ^2^Department of Medicine and Epidemiology, School of Veterinary Medicine, University of California, Davis, Davis, CA, United States; ^3^Department of Anatomy and Physiology, Institute of Computational Comparative Medicine (ICCM), College of Veterinary Medicine, Kansas State University, Manhattan, KS, United States; ^4^Department of Population Health and Reproduction, School of Veterinary Medicine, University of California, Davis, Davis, CA, United States; ^5^Department of Animal Science, College of Agricultural and Environmental Sciences, University of California, Davis, Davis, CA, United States; ^6^USDA-ARS Biosciences Research Laboratory, Fargo, ND, United States

**Keywords:** flunixin meglumine, subcutaneous, goat, pharmacokinetics, milk, withdrawal interval

## Abstract

**Introduction:** The study objectives were to estimate plasma flunixin (FLU) pharmacokinetic parameters and milk depletion profiles for FLU and its metabolite (5-hydroxy flunixin; 5-OH) after subcutaneous (SC) and intravenous (IV) administration of single and multiple flunixin meglumine (FM) doses to non-lactating (nulliparous and pregnant does) and lactating dairy goats. Analytical methods (ELISA and UPLC-MS/MS) for quantifying plasma FLU concentrations were compared. The final objective was to use regulatory (FDA and EMA) methods to estimate milk withdrawal intervals following extra-label drug use in goats.

**Methods:** FM was administered IV and SC to commercial dairy goats at 1.1 mg/kg for single and multiple doses. Plasma and milk samples were analyzed for FLU and 5-OH via UPLC-MS/MS. Plasma samples were also analyzed for FLU concentrations via ELISA. Using statistical approaches recommended by regulatory agencies, milk withdrawal intervals were estimated following FM extra-label use.

**Results:** Following IV administration of a single FM dose, clearances were 127, 199, and 365 ml/kg/h for non-lactating (NL) pregnant does, NL nulliparous does, and lactating dairy does, respectively. Following multiple SC doses, clearance/*F* was 199 ml/kg/h for lactating does. After IV administration of a single FM dose, terminal elimination half-lives were 4.08, 2.87, and 3.77 h for NL pregnant does, NL nulliparous does, and lactating dairy does, respectively. After multiple SC doses, the terminal elimination half-life was 3.03 h for lactating dairy does. No significant differences were noted for samples analyzed by UPLC-MS/MS or ELISA. Milk withdrawal intervals ranged from 36 to 60 h depending on the regulatory statistical method and dosage regimen.

**Conclusions:** Subcutaneous administration of FM to goats results in similar plasma pharmacokinetic parameters as IV administration. ELISA analysis is an alternative method to UPLC-MS/MS for quantifying FLU concentrations in caprine plasma samples. Following FM extra-label administration to dairy goats, clinicians could consider 36–60 h milk withdrawal intervals.

## Introduction

According to the United States Department of Agriculture (USDA), there are ~2.62 million goats used for meat, milk, and mohair production in the USA (1). Simultaneously, the demand for goat products is increasing as evidenced by the 19% increase in the number of dairy goats from 2011 to 2019 (2, 3). Despite this rapid growth of the US goat industry, there are currently no non-steroidal anti-inflammatory drugs (NSAIDs) approved by the FDA for goats. Instead, NSAIDs, such as flunixin meglumine (FM), are often used in an extra-label manner in the USA, as allowed by the Animal Medicinal Drug Use Clarification Act (4) and its implementing regulations published at Title 21, Code of Federal Regulations, Part 530 (5).

The use of NSAIDs is common in livestock species for treatment and/or control of endotoxemia, pyrexia, inflammation, and pain. These medications act by inhibiting the cyclooxygenase enzyme and the arachidonic acid cascade resulting in decreased production of prostaglandins and thromboxanes (6). As one of the most commonly used NSAIDs in food animal practice in the USA, FM has FDA approval for the treatment of endotoxemia and inflammation in cattle and swine when administered by IV (cattle) or intramuscular (IM; swine) routes. Examples of extra-label indications for administration of FM to goats include the treatment of pain, pyrexia, mastitis, and endotoxemia (7–12). Practical use of FM becomes problematic for veterinarians treating minor food-producing species (such as dairy goats) as they must often extrapolate milk withholding intervals based on cattle data in order to avoid drug residues in caprine milk. The extra-label use of FM can result in prolonged detectable drug residues in animal-derived food products such as veal destined for human consumption (13). Drug residue problems are not limited to meat, as FLU metabolite residues found in bovine milk are some of the most common violations (4). The problematic nature of this situation is furthered as there are no published goat studies that have evaluated the pharmacokinetics (PK) of subcutaneously (SC) administered FM, despite its common use. Additionally, no studies have performed preliminary investigations of tissue reactions when FM is administered SC to goats. Administration routes other than IV may be problematic in some species, as evidenced by the potential adverse effects of clostridial myositis linked to when FM is administered intramuscularly to horses (14).

Several formulations of FM have been studied in food-animal species. The use of FM formulated as granules has been evaluated in cattle (6, 15, 16) and goats (17). A dilution of the injectable formulation of FM has also been evaluated in chickens (18). In cattle, oral FM has been studied with respect to effects on prostaglandin metabolites (15), luteolysis (6), and response to endotoxemia (16). Another formulation of FM available for cattle is the transdermal suspension, which has an FDA-approved label indication for minimizing pain associated with foot rot following single transdermal application (19).

Several published studies that evaluated the PK of orally administered injectable formulations of FM have shown oral administration to be similar to injectable routes in cattle (6, 17), chickens (18), and, to a certain extent, goats (17). However, to date, no published studies exist that provide data for a recommended milk withdrawal interval when an injectable formulation of FM is administered SC in goats. This study aimed to determine plasma PK parameters for single and multiple SC and IV injections of FM to non-lactating and lactating dairy goats and to generate data on which to estimate milk withdrawal intervals following extra-label drug use.

## Methods

### Experimental Animals

**Figure 1:**
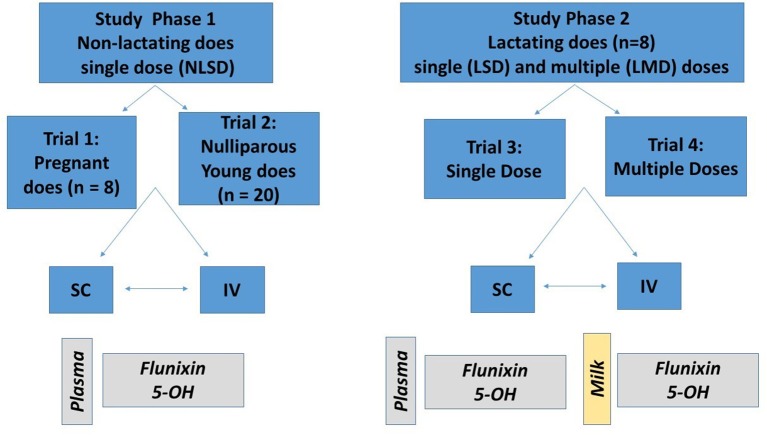
Description of study design. Study phase 1 (left) consisted of single dosing of 1.1 mg/kg of flunixin meglumine (FM) intravenously (IV) and subcutaneously (SC) to eight pregnant non-lactating does (trial 1) and 20 nulliparous does (trial 2). Study phase 2 (right) consisted of single (trial 3) and multiple (trial 4) dosing of 1.1 mg/kg FM to eight lactating does. For each trial, half of the animals were randomly assigned to receive either an IV or SC treatment; after a washout period, the opposite treatment was administered. For study phase 1, plasma concentrations of flunixin (FLU) and 5-hydroxy flunixin (5-OH) were determined. For study phase 2, plasma and milk concentrations of FLU and 5-OH were determined.

This project was approved by the Institutional Animal Care and Use Committee of the University of California-Davis. The project was divided into two study phases, with Phase 1 (trial 1 and trial 2) investigating the PK of a single dose of FM administered IV or SC to non-lactating dairy goats (pregnant and nulliparous does), and Phase 2 (trial 3 and trial 4) investigating the PK of single or multiple doses of FM administered IV or SC to lactating dairy goats. Goats were dosed FM at 1.1 mg/kg based on the low end of the bovine dosing range (1.1–2.2 mg/kg). Each study phase utilized a crossover study design based on administration routes as shown in Figure 1. All animals were determined to be healthy on the basis of physical examination with no evidence of mastitis. Physical examinations were performed before the studies were started and before and after each crossover. Animals were visually assessed daily for appetite, general appearance, and manure consistency. During acclimation, treatment, and washout periods, goats were housed outdoors in a group pen, and provided water *ad libitum*. The goats were fed alfalfa hay and grain diet that either met or exceeded the National Research Council requirements for maintenance of goats at their respective production status. The herd protocol included twice annual vaccinations against diseases associated with the following Clostridial agents: *chauvoei, septicum, haemolyticum, novyi, tetani*, as well as *perfringens types C & D* (Covexin 8^TM^, Merck Animal Health Kenilworth, New Jersey, USA). None of the animals selected for the study had a history of FM administration within 14 days of the start of the study.

### Phase 1 (Trials 1 and 2). PK of a Single Dose (SD) of FM Administered IV or SC to Non-lactating Dairy Goats (Nulliparous and Pregnant Does)

Healthy non-lactating dairy goats (*n* = 28) ranging in age from 1 to 9 years and weighing 43–99.5 kg were enrolled at two different study sites. Both pregnant and nulliparous animals were included. Dairy goat breeds used in the study included Toggenburg, Saanen, Oberhasli, LaMancha, and Alpine. The animals were housed in their normal location and had free access to hay and water throughout the study. The goats were not on any other medications during the study period.

For trial 1, eight non-lactating, pregnant, commercial dairy goats weighing 76 ± 11 kg (range: 64–96 kg) and aged 3.75 ± 3.33 years (range: 1–9 years) were housed at a seedstock production operation (Capay Valley, CA, USA). For trial 2, 20 non-lactating, nulliparous commercial dairy goats weighing 44.85 ± 7.35 kg (range: 32.5–61 kg) and aged 0.71 ± 0.04 years (range: 0.66–0.79 years) were used; these goats were housed at the University of California, Goat Teaching and Research Facility, Davis, CA.

### Phase 2 (Trials 3 and 4). PK of Single Dose (SD) or Multiple Doses (MD) of FM Administered IV or SC to Lactating Dairy Goats

For Phase 2, 8 lactating commercial dairy goats (4 Alpine, 4 La Mancha) weighing 85 ± 10 kg (range, 75–95 kg) and aged 3.75 ± 1.54 years (range: 2–6 years) were used; these goats were housed at the University of California, Goat Teaching and Research Facility, Davis, CA. Phase 2 was divided into a single-dose trial (trial 3) and a multiple-dose trial (trial 4). Daily milk production for the goats on the day prior to the start of this study was 5.21 ± 0.8 kg. Lactation status included the following: 1st lactation (2 goats), an extended first lactation (1 goat), 2nd lactation (2 goats), a 4th lactation (1 goat), and 5th lactation (2 goats). At the beginning of trial 3, the goats averaged 111 days in milk (range 49–441 days).

Injection sites following SD SC administration (trial 3) and MD SC administration (trial 4) were assessed according to a scoring system established for this project. Injection sites were clipped before FM administration and a permanent marker was used to identify each site for follow-up observation and scoring. For each doe, the injection site was monitored by the same investigator (JSS) daily during the first 10–14 days following FM administration and then several days prior to the start of the next crossover. The injection sites were subjectively evaluated by visual assessment and palpation for the presence or progression of swelling, heat, redness, or signs of pain, and these adverse reactions were recorded when evident. Injection sites were scored on a 5 point system that utilized four criteria: swelling, heat, redness, or pain. Daily scoring was assigned to each doe, with 1 point being awarded for the presence of swelling <2 cm in diameter and 2 points awarded for the presence of swelling >2 cm in diameter. One point was also awarded individually if heat, redness, or signs of pain were present, and a score of 0 would be awarded for each category if absent. With this system, a score of 5 points would be awarded for an injection site reaction with >2 cm swelling (2 points) that also exhibited heat (1 point), redness (1 point), and signs of pain (1 point). Similarly, an injection site with no swelling, heat, redness, or pain would receive a score of 0.

### Experimental Design and Sample Collection

#### Phase 1 (Trials 1 and 2). PK of a Single Dose (SD) of FM Administered IV or SC to Pregnant (Trial 1) and Nulliparous (Trial 2) Non-lactating (NL) Dairy Goats

For trials 1 and 2, goats were randomly assigned to receive FM (Banamine™, Merck Animal Health, Madison, NJ, USA) at a dose of 1.1 mg/kg either IV or SC. Single-dose injections were given SC in the mid-thoracic region caudal to the elbow. A crossover design was used such that goats were given FM by the alternate route (either IV or SC) after a minimum 28-day washout period. Ten-milliliter blood samples were collected by jugular venipuncture into heparinized tubes (BD vacutainer, BD, Franklin Lakes, NJ, USA). Samples were drawn at 0, 5, 10, 15, 30, and 45 min and 1, 1.5, 2, 4, 6, 8, 12, 18, 24, 30, 32, 36, and 48 h. Samples were drawn on the opposite jugular vein than where the drug was administered.

#### Phase 2 (Trials 3 and 4). PK of Single Dose (SD; Trial 3) or Multiple Doses (MD; Trial 4) of FM Administered IV or SC to Lactating Dairy Goats

##### Trial 3 (Single IV or SC dose; lactating does)

Goats were randomly assigned to receive FM at a dose of 1.1 mg/kg either via IV (*n* = 4 goats) or SC (*n* = 4 goats) administration. Single-dose injections were given SC in the mid-thoracic region caudal to the elbow. A crossover design was used such that goats were given FM by the alternate route (either IV or SC) after a minimum 14-day washout period. Prior to initiating FM treatment, a sample of milk and whole blood was collected. Each doe's udder was completely milked out prior to drug administration and sampling. Potential injection sites were also scored as described above. Goats were administered FM at time zero, and then blood samples were collected via direct venipuncture at 5, 10, 15, 30, 45, and 60 min after administration, with additional samples being collected at 2, 4, 6, 8, 12, 18, 24, 30, 36, 48, 60, and 72 h after administration. Milk samples were collected via hand milking at 1, 2, 4, 6, 8, and 18 h after administration. Milk samples were also collected by machine milking (Waikato Milking Systems^TM^, Hamilton, NZ) at 12, 24, 36, 48, 60, 72, 84, 96, 108, and 120 h after administration.

##### Trial 4 (multiple IV or SC doses; lactating does)

Goats were maintained in their same assigned administration-based treatment groups as for trial 3. For the multiple-dose study, FM treatments were alternated, with evening treatments administered on either the right side of the animal (SC) or the right jugular vein, and morning treatments administered on the left side of the animal (SC) or the left jugular vein. For the does assigned to the SC treatments, three distinct sites on the left and right side of the thoracic body wall caudal to the elbow were used for injection and were identified with permanent markers. In order to evaluate injection site reactions, pre-identified injection sites were used only once for FM administration. These sites were monitored for injection site reactions as described previously. A crossover design was used such that goats were administered FM by the alternate route (either IV or SC) after a minimum 14-day washout period. Trial 4 (multi-dose lactating does study) was conducted 15 weeks after the conclusion of trial 3 (single-dose lactating does study). Prior to initiating FM treatment, a sample of milk and blood was collected. Each doe's udder was completely milked out prior to drug administration and sampling. Injection sites were also evaluated as described above. Goats were administered a treatment regimen of FM (1.1 mg/kg) every 12 h for six treatments. The sample collected immediately prior to the administration of the sixth treatment marked the time 0. Whole-blood samples were collected by direct venipuncture at 5, 10, 15, 30, 45, and 60 min after administration, with additional samples being collected at 2, 4, 6, 8, 12, 18, 24, 30, 36, 48, 60, and 72 h after the last FM administration. Milk samples were collected via hand milking (complete evacuation of the udder) at 1, 2, 4, 6, 8, and 18 h after administration, with samples being collected by milking machine at 12, 24, 36, 48, 60, 72, 84, and 96 h after administration. For the first crossover of the multi-dose lactating does study, the second dose (12 h) pre-treatment milk samples were lost due to a sampling error.

### Sample Processing

For each sampling time point, whole-blood samples were stored on ice and were then centrifuged at 2,000 × *g* for 10 min. Plasma samples were stored frozen (−80°C) until analyzed.

Milk samples were agitated three times following collection and then placed into sample tubes and frozen (−20°C) until sample analysis.

### Quantifying FLU and 5-OH Metabolite Concentrations in Plasma and Milk Samples Using Ultra-Performance Liquid Chromatography With Mass Spectrometric Detection

Plasma and milk FLU and 5-hydroxy flunixin (5-OH) concentrations were quantified based on the method by Buur et al. (20), with minor modifications. Flunixin, flunixin-d3, and 5-hydroxy flunixin were VETRANAL™ analytical grade (≥99.5%; Fluka, St. Louis, MO, USA) and 5-hydroxyflunixin-d3 was analytical grade (95%; Santa Cruz Biotechnology, Dallas, TX, USA). Individual stock solutions of each were made by dissolving 1 mg in 1 ml of methanol. The mixed working solution for the internal standards was made by diluting 50 μl of each to 5 ml. Deuterated forms of FLU and 5-OH were used as internal standards. Samples were acidified with phosphoric acid and centrifuged. The supernatant was further extracted on a Waters Oasis MCX cartridge (1 ml, 30 mg). Samples were eluted with methanol:ammonium hydroxide (90:10, v/v), evaporated to dryness under nitrogen reconstituted with acetonitrile:water (40:60, v/v), and filtered with a 0.2-μm PVDF syringeless filter device.

Analysis was carried out on an Acquity UPLC (Waters) coupled to a Thermo TSQ Quantum Discovery Max tandem quadrupole mass spectrometer with a heated electrospray ionization source operated in the positive ion mode. The column was an Acquity UPLC HSS T3 (1.8 μm, 2.1 × 100 mm) maintained at 30°C. The mobile phase was 0.1% acetic acid:acetonitrile (32:68) at a flow rate of 0.4 ml/min. Ions were monitored in the selected reaction monitoring mode with transitions of 297–279 for FLU, 300–282 for flunixin-d3, 313–295 for 5-OH, and 316–298 for 5-OH flunixin-d3.

Additionally, samples from nine randomly selected does from study phase 1 were collected in crossover fashion after both IV and SC administration and analyzed via ELISA for FLU concentrations for agreement with UPLC-MS/MS results.

### Quantifying FLU and 5-OH Concentrations in Plasma and Milk Samples Using Enzyme-Linked Immunoassay (ELISA) Method

The FLU antibody-coated plate, horseradish peroxidase conjugate, and substrate provided by the manufacturer were used to quantify the FLU in goat plasma. The assay procedure followed those described by Shelver et al. (21) for cattle plasma with the exception of the calibration curves and were generated from control goat plasma dissolved in 50 mM phosphate buffer, pH 6.8, 1:1 (v/v). The calibration points included 0. 0.3, 0.5, 1, 3, 10, 30, 100, and 300 ng/ml fitted to a four-parameter logistic equation; the unknown concentrations of flunixin free acid in test samples were computed from the standard curve and adjusted for the dilution factor. If a sample concentration exceeded the highest calibration point, the sample was diluted using blank plasma: 50 mM phosphate buffer 1:1 (v/v) as diluent. The limit of detection (LOD), based on averaged inhibition concentration to reduce 10% of absorbance (IC_10_) and adjusted for the dilution factor, was 0.16 ng/ml. The working range based on IC_15_ to IC_85_ was 0.32–48.6 ng/ml.

### PK Analysis

PK analysis of time vs. total FLU and 5-OH plasma concentration data was completed using a noncompartmental model (Phoenix WinNonlin 8.0, Certara, Princeton, NJ, USA). Time vs. concentration figures for FLU and 5-OH were produced via a commercial program (GraphPad Prism 8.0.2, GraphPad Software, Inc., La Jolla, CA, USA).

Standard PK parameters were generated for individual does, as follows:

- Maximum observed FLU concentration (μg/ml), Obs *C*_max_;
- Last observed FLU concentration (μg/ml), Obs *C*_last_;
- Time to last observed FLU concentration (min), Obs *T*_last_;
- Area under FLU concentration–time curve from time zero to infinity [(ng/ml)^*^h], AUC_inf_;
- Area under FLU concentration–time curve from time zero to last measurement [(ng/ml)^*^h], AUC_last_;
- Area under FLU concentration–time curve extrapolated (%), AUC_*%ext*_;
- Flunixin mean residence time (MRT; h),
- MRT = AUMC_inf_/AUC_inf_, where AUMC_inf_ is the area under the first-moment curve from time zero to infinity [(ng/ml)^*^h^2^]. Area parameters (AUC, AUMC) were calculated using the log-linear trapezoidal rule.
- FLU, terminal elimination half-life (*T*_1/2λ*z*_, h)
- *T*_1/2λ*z*_ = ln (2)/λ_z_, where λ_z_ is the slope of the terminal phase of the natural logarithm of concentrations vs. time curve
- FLU systemic clearance (ml/h/kg), CL = Dose/AUC_inf_;
- Volume of distribution (ml/kg) of flunixin during the elimination phase,
- *V*_area_ = Dose/(AUC_inf_ × λ_z_); (*V*_z_)
- Volume of distribution (ml/kg) of flunixin at steady state,
- *V*_ss_ = CL × MRT

For FLU, the extraction ratio (*E*_body_) was calculated as previously described (22–24) with:

*E*_body_ = Systemic clearance/Cardiac output (1)

First, we calculated for each individual doe, and then combined them for a mean value, with the doe cardiac output described by Toutain et al. (22), as follows:

Cardiac output = 180 × BW(kg)^−0.19^ (2)

Additionally, the bioavailability (*F*) of flunixin following SC administration was calculated as follows: (AUC_INFSC_)/(AUC_INFIV_).

### Statistical Analysis

Within time point, mean plasma concentrations as determined after ELISA or UPLC–MS/MS analysis were compared using the Student's *t* test after testing for equal variance. Type I statistical error, common with multiple comparisons, was minimized by comparing only means between analytical method within dose route and time point. A similar procedure was used for the comparison of within dose route concentrations calculated from ELISA or UPLC-MS/MS data.

For determination of the effect of age and reproductive status on the PK of IV and SC FLU PK, the PK parameters from trials 1 and 2 were compared. Data distributions for all PK parameters were assessed for normality by Shapiro–Wilk tests. Comparisons between the two experimental groups were performed via unpaired *t*-tests for normally distributed parameters and Mann–Whitney tests for nonparametric parameters via a commercial program (GraphPad Prism 8, GraphPad Software, Inc, La Jolla, CA, USA) as previously described (25, 26).

For all statistical tests, *P* < 0.05 was considered significant.

### Estimation of Milk Withdrawal Interval

Milk withdrawal intervals for FLU after differing administration regimens were calculated using European Medicines Agency (EMA)'s WTM 1.4 software and US FDA's “reschem” R package. The WTM 1.4 software is an updated computerized version of a harmonized approach for the calculation of withdrawal periods for milk throughout the European Union. This program was initially released in 2000 and later updated and adopted in 2002 by the Committee for Veterinary Medicine Products (CVMP) of EMA. Several calculation methods are available in the WTM 1.4 software, including the safe concentration from linear regression (SCLR) method and the time-to-safe-concentration (TTSC) method. The TTSC method is not applicable to the present data because FLU has a zero tolerance in goat milk; thus, the tolerance is operationally equivalent to the LOD of 0.4 ng/ml for the marker residue 5-OH in milk, which is lower than all the quantifiable concentrations. Therefore, the SCLR calculation method was used to analyze the present data. The limit of quantification (LOQ) of the marker residue 5-OH in milk was 0.9 ng/ml. The CV of the assay was 5.7%. For FDA's “reschem” R package, it requires a minimal of 10 animals and triplicate measurements for each time point. However, the present study only included eight animals and only one measurement was performed for each sample at each time point. In order to satisfy the data format for using the “reschem” package, Crystal Ball (Version 11.1.2.4, Oracle Corporation, Redwood Shores, CA) was used to perform Monte Carlo simulation based on the mean concentration (i.e., the individual measured value) and the intra-assay CV of 3.2% to generate two replicate values. Also, the mean concentration and standard deviation of the studied animals at each time point were calculated and used to run Monte Carlo simulation to generate additional virtual animals to satisfy the requirement for at least 10 animals.

## Results

### Goat Health and Injection Site Reactions

No adverse effects (sedation, seizures, vomiting, diarrhea, or respiratory compromise) were observed following IV or SC FM administration. For trial 3, injection site scores were 1 and 2 in six and two goats, respectively. For trial 4, injection site scores were 1 and 2–3 in 5 and 3 does, respectively. None of the does had an injection reaction that persisted beyond the washout period to the next study or exit physical examination.

The LOD for FLU was 0.1 and 0.3 ng/ml in plasma and milk, respectively, and 0.3 and 0.4 ng/ml for 5-OH. The LOQ for FLU was 0.5 and 0.9 ng/ml in plasma and milk, and for 5-OH, it was 0.8 and 0.9 ng/ml. Average intra- and inter-assay variability, as measured by relative standard deviation, in plasma was 4.3 and 5.2% for FLU and 4.3 and 5.7% for 5-OH. In milk, the intra- and inter-assay variability was 3.4 and 4.7% for FLU and 3.2 and 5.7% for 5-OH. Average recoveries were 100.0% for both FLU and 5-OH in plasma and 99.1 and 99.6%, respectively, in milk.

### Study Phase 1

Following IV administration of a single dose of FM, mean FLU concentration (Obs *C*_max_) was 9.972 ± 2.596 (pregnant NL does) and 9.613 ± 2.737 μg/ml (nulliparous NL does) after single dosing. Following SC single dosing, the mean observed peak plasma concentration (Obs *C*_max_) was 2.182 ± 0.255 (pregnant NL does) and 2.495 ± 1.234 (nulliparous NL does) μg/ml and the observed time to maximum concentration (Obs *T*_max_) occurred 1.41 ± 0.38 and 0.90 ± 0.42 h after SC administration, respectively.

Table 1 summarizes plasma PK parameters after IV and SC single dose administration of FM to pregnant non-lactating does (trial 1) and nulliparous non-lactating does (trial 2).

**Table 1:** Non-compartmental pharmacokinetic parameters (mean, SD) of flunixin and 5-hydroxy flunixin (5-OH) after a single intravenous (IV) and subcutaneous (SC) flunixin meglumine dose (1.1 mg/kg) administered to non-lactating dairy does.

		**Trial 1**	**Trial 1**	**Trial 2**	**Trial 2**
**Parent drug or metabolite**	**PK parameter**	**Mean ± SD (IV)**	**Mean ± SD (SC)**	**Mean ± SD (IV)**	**Mean ± SD (SC)**
Flunixin	*C*_max_ (μg/ml)	9.972 ± 2.596	2.182 ± 0.255	9.613 ± 2.737	2.495 ± 1.234
	*T*_max_ (h)	NA	1.41 ± 0.38	NA	0.90 ± 0.42
	*T*_1/2*Z*_ (h)	2.17 ± 2.07	4.08 ± 22.5	3.13 ± 2.26	2.87 ± 3.89
	Z (h^−1^)	0.21 ± 0.24	0.06 ± 0.17	0.18 ± 0.09	0.15 ± 0.15
	AUC_inf_ (h*μg/ml)	9.880 ± 2.327	8.806 ± 1.762	6.429 ± 2.187	5.780 ± 1.828
	AUC_%ext_ (%)	0.017 ± 0.072	0.12 ± 6.38	0.025 ± 0.062	0.023 ± 0.078
	*V*_z_, *V*_z_/*F* (ml/kg)	444 ± 303	1,071 ± 2,978	903 ± 594	1,001 ± 1,634
	Cl, CL/*F* (ml/kg/h)	114.0 ± 24.4	127 ± 26.4	182 ± 83.7	199 ± 75.5
	AUMC_inf_ (h*h*μg/ml)	27.205 ± 15.539	51.310 ± 116.135	11.710 ± 5.056	17.222 ± 7.269
	MRT_inf_ (h)	2.74 ± 0.80	5.07 ± 9.48	1.91 ± 0.03	3.03 ± 0.77
	*E*_body_	1.44 ± 0.29	–	2.08 ± 0.98	–
	*F* (%)	–	89.0 ± 5.0	–	94.0 ± 3.3
5-OH	*C*_max_ (μg/ml)	0.1006 ± 0.0203	0.0493 ± 0.0143	0.1476 ± 0.0539	0.0668 ± 0.0267
	*T*_max_ (h)	0.23 ± 0.45	1.55 ± 0.35	0.16 ± 0.09	1.08 ± 0.42
	*T*_1/2*Z*_ (h)	4.18 ± 1.1	5.41 ± 6.88	3.64 ± 2.47	3.67 ± 3.87
	Z (h^−1^)	0.16 ± 0.04	0.09 ± 0.05	0.15 ± 0.09	0.14 ± 0.1
	AUC_inf_ (h*μg/ml)	0.244 ± 0.083	0.243 ± 0.072	0.241 ± 0.080	0.208 ± 0.056
	*V*_z_, *V*_z_/*F* (ml/kg)	29,190 ± 13,876	43,015 ± 41,692	28,131 ± 21,109	33,595 ± 30,352
	Cl, CL/*F* (ml/kg/h)	4,718 ± 1,393	4,665 ± 1,395	4,792 ± 1,678	5,549 ± 2,755
	AUMC_inf_ (h*h*μg/ml)	1.013 ± 0.533	1.547 ± 1.237	0.700 ± 0.339	0.835 ± 0.342
	MRT_inf_ (h)	4.25 ± 1.01	6.31 ± 2.48	2.94 ± 0.78	4.09 ± 1.27

*AUC_inf_, area under the concentration curve from time 0 to infinity; AUC_%ext_ (%), area under the concentration curve extrapolated; AUMC_inf_, area under the moment curve from time zero to infinity; C_max_, maximum concentration observed; CL, observed clearance; MRT_inf_, mean residence time; E_body_, Extraction ratio; F, bioavailability; T_1/2Z_, half-life of terminal elimination; T_max_, observed time to maximum concentration; V_z_, volume of distribution; Z, terminal elimination constant*.

*Trial 1 consisted of 8 healthy pregnant non-lactating does. Trial 2 consisted of 20 nulliparous non-lactating does*.

**Figure 2:**
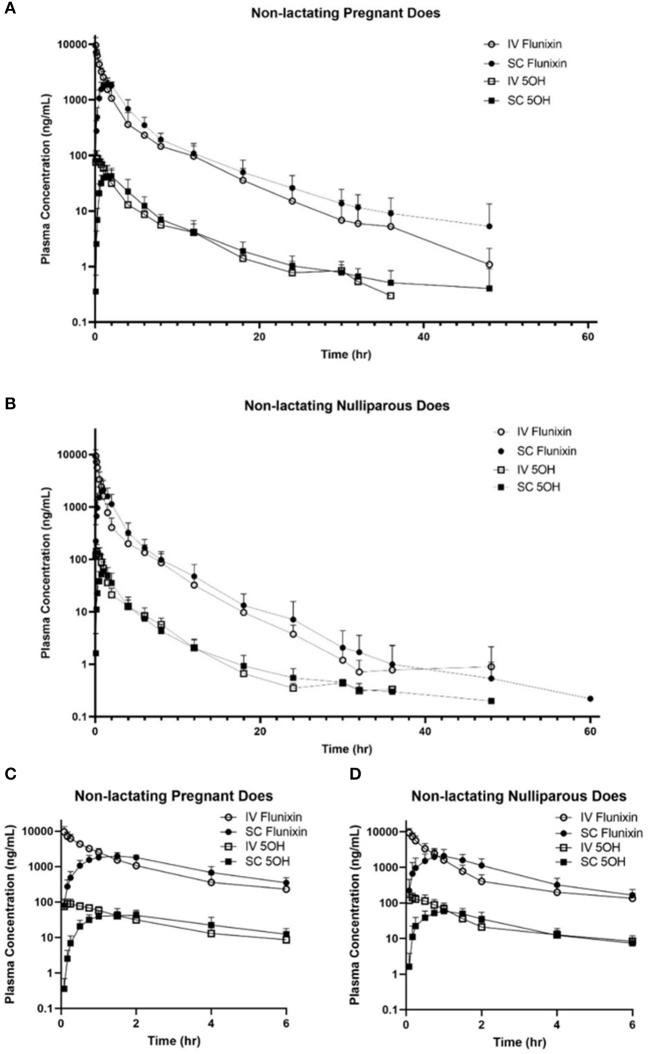
**(A,B)** Mean plasma flunixin (FLU) and 5-hydroxy flunixin (5-OH) concentration (logarithmic scale) vs. time profiles for **(A)** pregnant non-lactating (*n* = 8; trial 1) and **(B)** nulliparous non-lactating (*n* = 20; trial 2) dairy does following intravenous (IV) and subcutaneous (SC) single dose administration of 1.1 mg/kg of flunixin meglumine (FM). This study had a crossover design. Each trial was divided into 2 groups and randomly allocated to receive FM IV or SC. After a minimum 4-week washout period, each group received FM via the opposite administration route. Open circles and boxes correspond to plasma concentrations after IV FM administration; solid circles and boxes correspond to plasma concentrations after SC FM administration. **(C,D)** Mean plasma flunixin (FLU) and 5-hydroxy flunixin (5-OH) concentration (logarithmic scale) vs. time profiles for **(C)** pregnant non-lactating (*n* = 8; trial 1) and **(D)** nulliparous non-lactating (*n* = 20; trial 2) dairy does following intravenous (IV) and subcutaneous (SC) single dose administration of 1.1 mg/kg of flunixin meglumine (FM) focusing on the initial 6 h following drug administration. This study had a crossover design. Each trial was divided into two groups and randomly allocated to receive FM IV or SC. After a minimum 4-week washout period, each group received FM via the opposite administration route. Open circles and boxes correspond to plasma concentrations after IV FM administration; solid circles and boxes correspond to plasma concentrations after SC FM administration.

Figure 2 represents the time vs. plasma concentration profile for single-dose IV and SC administration of FM to non-lactating dairy does (trials 1 and 2). Figures 2A,B present the entire time vs plasma concentrate.on profile, whereas Figures 2C,D present the time vs. plasma concentration profile for the first 6 h after administration to focus on the differences in the early period after administration.

No significant differences were found on comparison of IV PK parameters between the groups of trials 1 and 2 for *C*_max_ (*P* = 0.9379) and *T*_1/2λ*z*_ (0.5845). Significant differences of IV PK parameters for groups 1 and 2 were as follows: CL (*P* = 0.0019), AUC _last_ (*P* = 0.0001), MRT (*P* = 0.0001), and *V*_z_ (*P* = 0.0238). For SC PK parameters, no significant differences were identified between trials 1 and 2 for *C*_max_ (*P* = 0.4872), *T*_1/2λ*z*_ (*P* = 0.3074), and *V*_z_ (*P* = 0.8557). Significant differences of SC PK parameters for groups 1 and 2 were as follows: *T*_max_ (*P* = 0.0179), CL/*F* (*P* = 0.0071), AUC_last_ (*P* = 0.0002), and MRT (*P* = 0.0012).

### Study Phase 2

Plasma PK parameters after IV and SC administration of single and multiple doses of FM in lactating dairy does are reported in Table 2. Following single IV administration, FLU concentration (Obs *C*_max_) was 7.235 ± 3.3252 μg/ml. After single SC dosing, FLU concentration (Obs *C*_max_) was 0.838 ± 0.2372 μg/ml at 1.28 ± 0.31 h post last administration. Following multiple IV administration, FLU concentration (Obs *C*_max_) was 7.691 ± 1.3452 μg/ml. After multiple SC dosing, the mean observed peak plasma concentration (Obs *C*_max_) was 1.434 ± 0.2237 μg/ml and the observed time to maximum concentration (Obs *T*_max_) occurred 1.17 ± 0.44 h after the last administration.

**Table 2:** Estimated non-compartmental pharmacokinetic parameters (mean ± SD) based on time vs. plasma flunixin and 5-OH concentration profiles after single (trial 3) and multiple (trial 4) doses of flunixin meglumine administered intravenously (IV) and subcutaneous (SC) at 1.1 mg/kg to eight healthy lactating dairy does.

		**Trial 3**	**Trial 3**	**Trial 4**	**Trial 4**
**Parent drug or metabolite**	**PK parameter**	**Mean ± SD (IV)**	**Mean ± SD (SC)**	**Mean ± SD (IV)**	**Mean ± SD (SC)**
Flunixin	*C*_max_ (μg/ml)	7.2346 ± 3.3252	0.8383 ± 0.2372	7.6909 ± 1.3452	1.4341 ± 0.2237
	*T*_max_ (h)	NA	1.28 ± 0.31	NA	1.17 ± 0.44
	*T*_1/2*Z*_ (h)	4.56 ± 2.70	3.77 ± 5.69	4.35 ± 2.43	3.03 ± 1.57
	Z (h^−1^)	0.122 ± 0.08	0.11 ± 0.13	0.13 ± 0.08	0.19 ± 0.09
	AUC_inf_ (h*μg/ml)	4.319 ± 1.350	3.059 ± 0.619	6.335 ± 0.993	5.672 ± 1.185
	AUC_%ext_ (%)	0.28 ±0.27	0.28 ± 0.26	0.057 ± 0.16	0.035 ± 0.053
	*V*_z_, *V*_z_/*F* (ml/kg)	1,945 ± 1,357	2,518 ± 3,295	1,222 ± 611	939 ± 499
	Cl, CL/*F* (ml/kg/h)	265 ± 78.4	365 ± 59.1	176 ± 29.4	199 ± 57.8
	AUMC_inf_ (h*h*μg/ml)	7.842 ± 1.767	11.790 ± 1.903	15.971 ± 6.142	22.503 ± 8.551
	MRT_inf_ (h)	1.83 ± 0.62	3.88 ± 0.61	2.38 ± 1.03	3.99 ± 0.86
	*E*_body_	3.55 ± 1.00	–	–	–
	*F* (%)	–	74.0 ± 20.0	–	–
5-OH	*C*_max_ (μg/ml)	0.1341 ± 0.0245	0.0552 ± 0.0185	0.1385 ± 0.0343	0.0605 ± 0.0153
	*T*_max_ (h)	0.25 ± 0.18	1.33 ± 0.42	0.19 ± 0.04	1.65 ± 0.26
	*T*_1/2*Z*_ (h)	3.65 ± 3.24	3.34 ± 1.05	3.68 ± 5.43	2.99 ± 3.23
	Z (h^−1^)	0.14 ± 0.09	0.19 ± 0.08	0.12 ± 0.09	0.16 ± 0.14
	AUC_inf_ (h*μg/ml)	0.2216 ± 0.0408	0.2372 ± 0.0673	0.2170 ± 0.0564	0.2631 ± 0.0780
	*V*_z_, *V*_z_/*F* (ml/kg)	30,209 ± 22,990	24,200 ± 10,441	32,388 ± 38,001	22,395 ± 34,303
	Cl, CL/*F* (ml/kg/h)	5,034 ± 894	4,785 ± 1,229	5,205 ± 1,196	4,384 ± 1,818
	AUMC_inf_ (h*h*μg/ml)	0.745 ± 0.251	1.007 ± 0.233	0.694 ± 0.306	1.129 ± 0.487
	MRT_inf_ (h)	3.34 ± 0.90	3.34 ± 0.89	3.10 ± 1.08	4.36 ± 1.13

*AUC_inf_, area under the concentration curve from time 0 to infinity; AUC_%ext_ (%), area under the concentration curve extrapolated; AUMC_inf_, area under the moment curve from time zero to infinity; C_max_, maximum concentration observed; CL, observed clearance; MRT_inf_, mean residence time; E_body_, Extraction ratio; F, bioavailability; T_1/2Z_, half-life of terminal elimination; T_max_, observed time to maximum concentration; V_z_, volume of distribution; Z, terminal elimination constant*.

*Trial 3: Lactating dairy does (n = 8). Trial 4: Lactating dairy does (n = 8)*.

**Figure 3:**
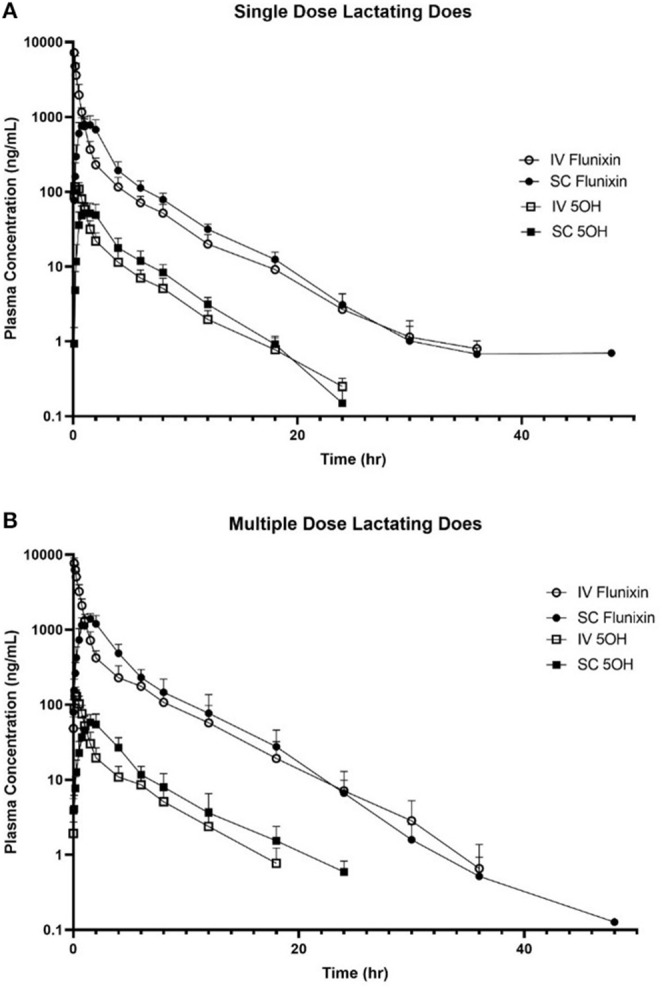
Mean plasma flunixin (FLU) and 5-hydroxy flunixin (5-OH) concentration (logarithmic scale) vs. time profiles for **(A)** single-dosed lactating (*n* = 8; trial 3) and **(B)** multiple-dosed lactating (*n* = 8; trial 4) dairy does following intravenous (IV) and subcutaneous (SC) administration of 1.1 mg/kg of flunixin meglumine (FM). This study had a crossover design. Each trial was divided into two groups and randomly allocated to receive FM IV or SC. After a minimum 2-week washout period, each group received FM via the opposite administration route. For the multiple-dose study, 6 doses of FM were administered over a 60-h period, with 1 dose q 12 h, with sampling commencing immediately after administration of the 6th dose. Open circles and boxes correspond to plasma concentrations after IV FM administration; solid circles and boxes correspond to plasma concentrations after SC FM administration.

Figure 3 is the time vs. plasma concentration curve for single and multiple doses of FM administered IV and SC to lactating dairy does.

**Figure 4:**
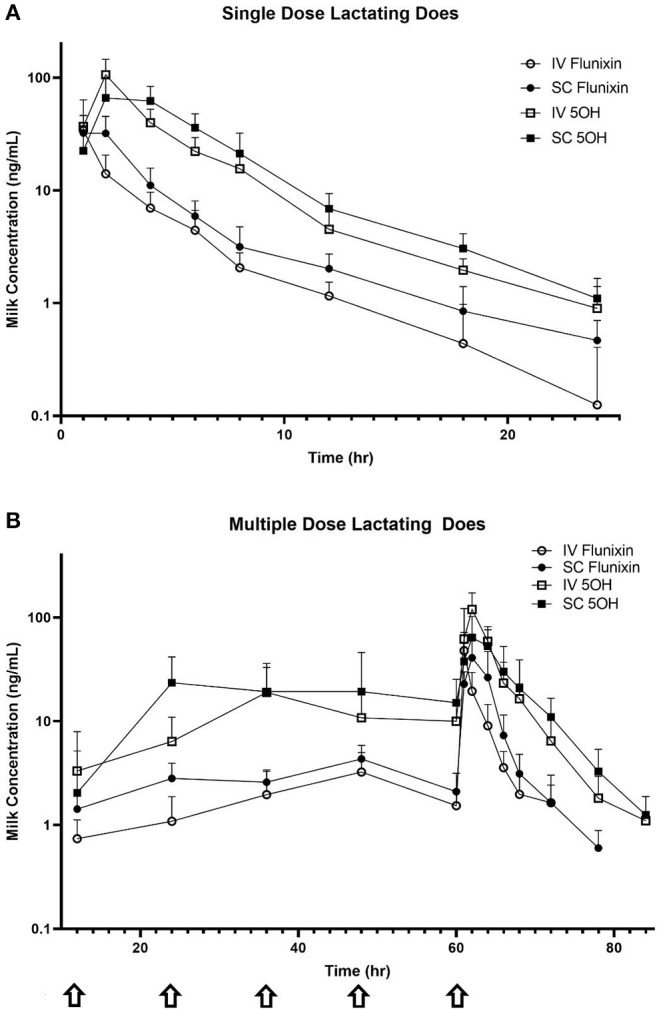
Mean milk flunixin (FLU) and 5-hydroxy flunixin (5-OH) concentration (logarithmic scale) vs. time profiles for **(A)** single-dosed lactating (*n* = 8; trial 3) and **(B)** multiple-dosed lactating (*n* = 8; trial 4) dairy does following intravenous (IV) and subcutaneous (SC) administration of 1.1 mg/kg of flunixin meglumine (FM). This study had a crossover design. Each trial was divided into two groups and randomly allocated to receive FM IV or SC. After a minimum 2-week washout period, each group received FM via the opposite administration route. For the multiple-dose study, 6 doses of FM were administered over a 60-h period, with one dose q 12 h, with sampling commencing immediately after administration of the 6th dose. Sampling commenced immediately after administration of the initial dose, and for the multiple-dose study, concentrations were measured immediately prior to administration of FM at 12, 24, 36, 48, and 60 h (arrows correspond to these dosing events). Open circles and boxes correspond to plasma concentrations after IV FM administration; solid circles and boxes correspond to plasma concentrations after SC FM administration.

Figure 4 is the time vs. milk concentration profile of FLU and 5-OH following single and multiple doses of FM administered IV and SC to lactating dairy does.

Reported extraction ratios were (mean ± SD) 1.44 ± 0.29 for pregnant NL does, 2.08 ± 0.98 for nulliparous NL goats, and 3.55 ± 1.00 for lactating does after single IV administration. Mean ± SD bioavailability was 89.0% for pregnant NL does, 94.0% for nulliparous NL goats, and 74.0% for lactating does after single SC administration.

### ELISA vs. UPLC-MS/MS Concentrations

Table 3 summarizes the FLU plasma concentrations quantified using ELISA and compared to UPLC-MS/MS determined concentrations. No statistically significant differences (*P* < 0.05) were identified for concentrations at any time point.

**Table 3:** Mean (± SD) flunixin plasma concentrations as a function of time as determined by immunochemistry (ELISA) or UPLC-MS/MS analysis in non-lactating does dosed (1.1 mg/kg bw of flunixin meglumine) by intravenous (*n* = 9) or subcutaneous (*n* = 9) injections.

**Intravenous administration**					**Subcutaneous administration**				
	**Immunochemical analysis**		**UPLC-MSMS analysis**			**Immunochemical analysis**		**UPLC-MSMS analysis**		
**Time**	**Mean ± SD**	**Range**	**Mean ± SD**	**Range**	***P***	**Mean ± SD**	**Range**	**Mean ± SD**	**Range**	***P***
**h**	**ng/ml**	**ng/ml**	**ng/ml**	**ng/ml**		**ng/ml**	**ng/ml**	**ng/ml**	**ng/ml**	
0.083	12,287 ± 4,254	5,688–19,593	10,529 ± 1,152	8,938–12,107	0.205	205 ± 236	25–803	305 ± 321	21–958	0.426
0.167	8,847 ± 2,673	4,912–13,525	7,524 ± 918	6,323–9,155	0.090	644 ± 477	128–1,638	929 ± 941	98–2,884	0.734
0.25	5,623 ± 2,524	1,936–8,590	6,138 ± 751	4,751–7,342	0.462	1,476 ± 1,223	191–3,928	1,335 ± 1,095	178–3,406	0.133
0.5	3,912 ± 983	2,814–5,734	4,187 ± 523	3,612–5,084	0.293	2,144 ± 1,155	552–4,104	1,850 ± 879	473–2,873	0.102
0.75	2,499 ± 1,031	1,071–4,317	2,677 ± 567	2,040–3,636	0.425	2,458 ± 947	1,119–3,801	2,146 ± 733	873–3,051	0.067
1	2,207 ± 775	1,222–3,552	1,933 ± 509	1,446–3,022	0.052	2,288 ± 662	1,600–3,133	2,066 ± 649	1,236–3,388	0.931
1.5	1,080 ± 601	393–2,241	979 ± 284	641–1,580	0.450	2,048 ± 805	841–3,020	1,755 ± 586	803–2,526	0.273
2	597 ± 204	299–880	520 ± 212	258–941	0.058	1,429 ± 742	395–2,703	1,396 ± 628	204–1,996	0.993
4	233 ± 76	82–322	217 ± 57	108–307	0.438	551 ± 566	113–1,930	513 ± 396	185–1,404	0.910
6	187 ± 72	49–281	186 ± 38	144–233	0.947	238 ± 167	88–502	242 ± 133	91–503	0.652
8	120 ± 31	68–158	120 ± 34	77–184	0.991	129.5 ± 48.2	67–203	129 ± 67	37–266	0.648
12	80 ± 55	30–176	62 ± 43	31–170	0.250	66 ± 38	19–147	64 ± 43	8.3–135	0.966
18	20 ± 19	8.2–66	22 ± 22	8.0–76	0.138	30 ± 25	4.3–86	25 ± 23	2.0–71	0.487
24	9.5 ± 12	2.6–36	9.3 ± 9.9	3–34	0.820	18 ± 17	1.2–47	17 ± 18	1.7–52	0.469
30^a^	4.5 ± 7.6	0.7–24	3.6 ± 5.0	0.5–16	0.445	13 ± 13^b^	0.5–32	9.2 ± 11^b^	0.3–26	0.5054
32^a^	3.3 ± 4.9^b^	0.3–13	2.8 ± 4.3	0.2–13	0.9685	9.5 ± 9.9^b^	0.6–25	8.3 ± 9.2^f^	0.1–21	0.813
36^a^	4.8 ± 3.9^c^	0.4–7.6	2.6 ± 3.4^d^	0.2–8.7	0.4002	9.0 ± 8.5^d^	1.3–24	9.1 ± 9.0^g^	0.11–24	0.9847
48^a^	2.2 ± 1.7^e^	0.9–3.4	1.6 ± 0.9^c^	0.9–2.5	0.5233	6.9 ± 11.4^d^	0.6–29	6.9 ± 9.9^h^	1.5–22	0.9994

a *Only values greater than limit of quantitation (LOQ) were used to calculate means*.

b *Means were calculated from 8 of 9 animals that had residue concentrations ≥ the LOQ*.

c *Means were calculated from 3 of 9 animals that had residue concentrations ≥ the LOQ*.

d *Means were calculated from 6 of 9 animals that had residue concentrations ≥ the LOQ*.

e *Means were calculated from 2 of 9 animals that had residue concentrations ≥ the LOQ*.

f *Means were calculated from 7 of 9 animals that had residue concentrations ≥ the LOQ*.

g *Means were calculated from 5 of 9 animals that had residue concentrations ≥ the LOQ*.

h *Means were calculated from 4 of 9 animals that had residue concentrations ≥ the LOQ*.

*P < 0.05 considered statistically significant*.

### Estimation of Milk Withdrawal Intervals

**Figure 5:**
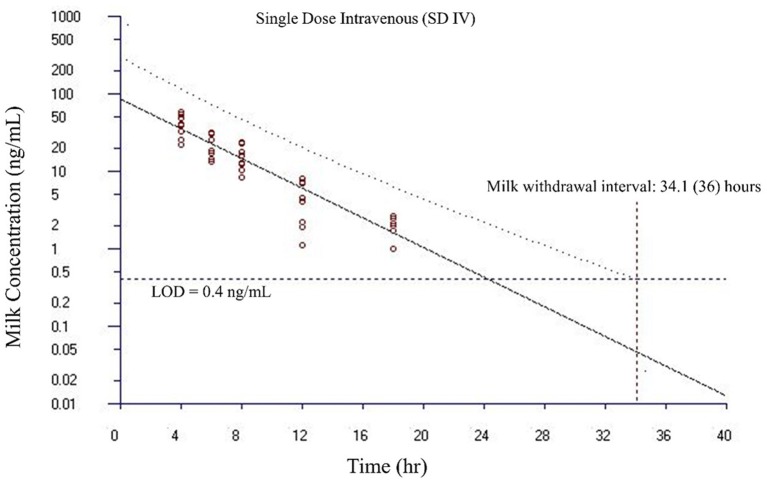
Estimated milk withdrawal interval for flunixin in lactating goats after single intravenous injection at 1.1 mg/kg. The calculation was based on the milk data of 5-hydroxy flunixin (i.e., the marker residue of flunixin in milk) using the European Medicines Agency's WTM 1.4 software (i.e., the EMA method). Since flunixin has a zero tolerance in goat milk, the tolerance or maximum residue limit was set to be operationally equivalent to the limit of detection (LOD) of 0.4 ng/ml for the marker residue 5-hydroxy flunixin in milk. The estimated milk withdrawal interval was rounded to the next milking interval (shown in parenthesis assuming a 12-h milking interval).

**Figure 6:**
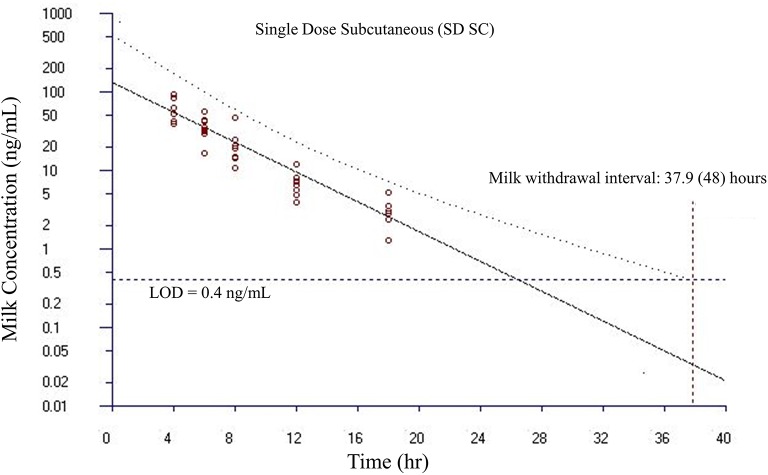
Estimated milk withdrawal interval for flunixin in lactating goats after single subcutaneous injection at 1.1 mg/kg. The calculation was based on the milk data of 5-hydroxy flunixin (i.e., the marker residue of flunixin in milk) using the European Medicines Agency's WTM 1.4 software (i.e., the EMA method). Since flunixin has a zero tolerance in goat milk, the tolerance or maximum residue limit was set to be operationally equivalent to the limit of detection (LOD) of 0.4 ng/ml for the marker residue 5-hydroxy flunixin in milk. The estimated milk withdrawal interval was rounded to the next milking interval (shown in parenthesis assuming a 12-h milking interval).

**Figure 7:**
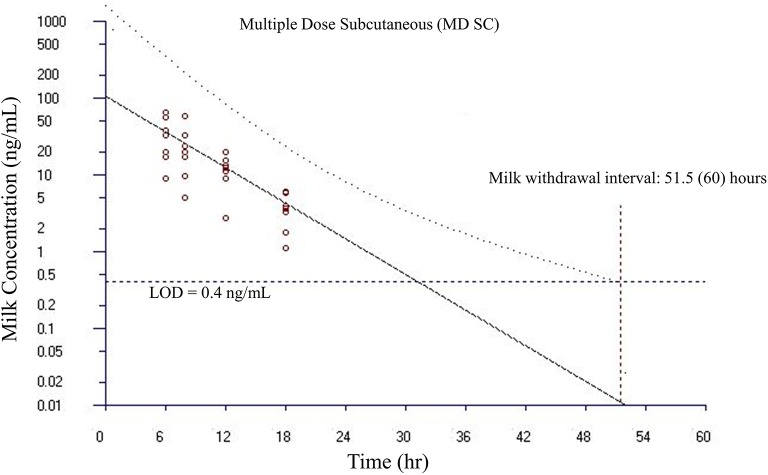
Estimated milk withdrawal interval for flunixin in lactating goats after multiple subcutaneous injections at 1.1 mg/kg every 12 h for 6 injections. The calculation was based on the milk data of 5-hydroxy flunixin (i.e., the marker residue of flunixin in milk) using the European Medicines Agency's WTM 1.4 software (i.e., the EMA method). Since flunixin has a zero tolerance in goat milk, the tolerance or maximum residue limit was set to be operationally equivalent to the limit of detection (LOD) of 0.4 ng/ml for the marker residue 5-hydroxy flunixin in milk. The estimated milk withdrawal interval was rounded to the next milking interval (shown in parenthesis assuming a 12-h milking interval).

Milk withdrawal intervals ranged from 36 to 60 h post last dose depending on the dosing paradigms and regulatory methods. The results are presented in Table 4. The milk withdrawal interval output figures from EMA's WTM 1.4 software for SD IV, SD SC, and MD SC are displayed in Figures 5–7, respectively. The MD IV dataset was not calculable using the EMA method.

**Table 4:** Estimated milk withdrawal intervals for lactating goats after single or multiple doses of flunixin meglumine administered intravenously or subcutaneously.

	**EMA method**	**FDA method**
Single IV administration at 1.1 mg/kg	34.1 h (36 h)	36 h
Single SC administration at 1.1 mg/kg	37.9 h (48 h)	36 h
Repeated IV administration at 1.1 mg/kg at 12-h intervals for 6 injections	NA	36 h
Repeated SC administration at 1.1 mg/kg at 12-h intervals for 6 injections^*^	51.5 h (60 h)	48 h

*The EMA method refers to the computerized version of the method (i.e., WTM 1.4 software) used to calculate withdrawal periods for milk developed by the Committee for Veterinary Medicinal Products (CVMP) of European Medicines Agency (EMA). The FDA method refers to the tolerance limit method (coded in the “reschem” R package) used to calculate milk withdrawal interval times developed by US Food and Drug Administration (FDA). The EMA method outputs the exact time when the drug concentration is below the tolerance (i.e., the maximum residue limit), and this time should be rounded to the next milking interval (shown in parenthesis assuming a 12-h milking interval). NA indicates that the withdrawal time is not calculable due to missing values or high variability of the data*.

* *Animal #8 in the repeated SC injection study was excluded because it was an apparent outlier*.

*Milk withdrawal intervals following extra-label drug use were estimated using EMA and FDA regulatory methods*.

## Discussion

In this study, we compared the PK parameters of IV and SC of FLU and 5-OH after FM administered to non-lactating and lactating dairy does of multiple ages. Additionally, milk FLU and 5-OH concentrations were determined after each treatment. Our present study is novel because we determined these parameters, including from SC injection, in lactating does grouped to mimic a commercial herd population, using a different route of administration, dose, as well as non-lactating goats of various ages and reproductive statuses. Additionally, FLU concentrations quantified using an ELISA method were compared to those using UPLC-MS/MS to determine if analytical method choice would result in similar FLU concentrations. The time vs. milk 5-OH FLU concentration data from our study were then used to estimate a milk withdrawal interval using statistical approaches to ensure that 95 or 99% of animals' milk will be free of residues 95% of the time.

Our study provided data for estimating plasma PK parameters following IV and SC administrations to pregnant non-lactating does (trial 1), non-lactating nulliparous does (trial 2), and lactating does (trials 3 and 4). Although FLU PK parameters for dairy goats (dosed at 2.2 mg/kg) were previously published (17), these parameters had been identified in dairy goats administered IV, IM, or orally (in granule form) FM, and in a population of 3-year-old goats of the Norwegian breed. That study determined their results using animals of the same age and did not evaluate SC administration of FM. In our study, absorption of FM was rapid after SC administration. The observed *C*_max_ determined for single-dose SC administrations in the groups of our study was 2.18 μg/ml (non-lactating pregnant does), 2.50 μg/ml (non-lactating nulliparous does), and 0.84 μg/ml (lactating does). These observed *C*_max_ values appear to be less than reported for intramuscular (6.1 μg/ml) administration as well more than reported for transdermal (0.134 μg/ml) and oral (1.2 μg/ml) administration in goats (with the exception of the *C*_max_ from the lactating group), although the transdermal study used a dosage of 3.3 mg/kg instead of 2.2 mg/kg (17, 27). The plasma *T*_1/2λ*z*_ of 4.08 h (pregnant NL does), 2.87 h (nulliparous NL does), and 3.77 h (lactating does) for SD SC treatment is consistent with what would be expected with other studies, as *T*_1/2λ*z*_ of 3.6 h (IV), 3.4 h (IM), 4.3 h (PO), and 43.1 h (transdermal) have been previously identified in caprine flunixin PK studies (17, 27). When compared to the existing literature, the plasma clearance (CL) of SD IV administered FM in the goats of our study of 114 ml/h/kg (pregnant NL does), 182 ml/h/kg (nulliparous, NL does), and 265 ml/h/kg (lactating does) appears to be higher than previously reported for goats as values of 110 and 67 ml/h/kg have been reported for adult Norwegian dairy goats and adult Boer goats, respectively (17, 27). Table 5 summarizes comparative PK parameters from our study with other caprine FM PK studies. It is important to note that PK parameters can be influenced by the lower LOQ, as recently demonstrated by IV fentanyl in calves (24). For our study the LOQ for flunixin was 0.5 and 0.9 ng/ml in plasma and milk, respectively, and for the 5-OH metabolite, it was 0.8 and 0.9 ng/ml, respectively. When comparing the quantification limits from our study to other studies of flunixin in goats, similarities of LOQs were noted for the meat goat study (0.5 ng/ml), but differed from the Norwegian dairy goat study (47 ng/ml) (17, 27).

**Table 5:** Goat plasma flunixin pharmacokinetic parameter comparisons described in the literature.

**Study**	**Population**	**n**	**Single dose route**	**Plasma elimination half-life (h)**	**Plasma maximum concentration (μg/ml)**	**Plasma time to maximum concentration (h)**	**Plasma clearance (ml/h/kg)**	**Bioavailability (*F*, %)**
Trial 1	Non-lactating	8	IV	2.17 ± 2.07	–	–	114.0 ± 24.4	–
Trial 1	Non-lactating	8	SC	4.08 ± 22.5	2.182 ± 0.255	1.41 ± 0.38	–	89.0 ± 5.0
Trial 2	Non-lactating	20	IV	3.13 ± 2.26	–	–	182 ± 83.7	–
Trial 2	Non-lactating	20	SC	2.87 ± 3.89	2.495 ± 1.23	0.90 ± 0.42	–	94.0 ± 3.3
Trial 3	Lactating	8	IV	4.56 ± 2.70	–	–	265 ± 78.4	–
Trial 3	Lactating	8	SC	3.77 ± 5.69	0.84 ± 0.24	1.28 ± 0.31	–	74.0 ± 20.0
Königsson et al. (17)	Adult Norwegian dairy goats	6	IV	3.6 (2.0–5.9)	–	–	110 (60–160)	–
Königsson et al. (17)	Adult Norwegian dairy goats	6	IM	3.4 (2.6–7.1)	6.1 (3.3–7.4)	0.37 (0.25–0.5)	–	79.0 (53–112)
Königsson et al. (17)	Adult Norwegian dairy goats	6	PO	4.2 (3.4–6.0)	1.2 (0.8–2.0)	3.5 (2.5–5.0)	–	58.0 (35–120)
Reppert et al. (27)	Adult meat goats	8	IV	6.03 ± 1.58	–	–	67.11 ± 14.74	–
Reppert et al. (27)	Adult meat goats	8	TD	43.12 ± 16.01	0.134 ± 0.042	11.41 ± 9.50	–	24.76 ± 6.5
Bublitz et al. (28)	5- to 8-month-old meat goats	5	IM	5.14 (3.9–15.7)	5.6 (4.7–7.6)	0.5 (0.25–0.5)	–	–

*This table describes study population, as well as dose route, plasma elimination half-life, time to maximum plasma concentration, plasma clearance, and bioavailability. Data are described with either standard deviation (±SD) or range (X–Y) depending on how this information was reported by the individual study*.

In cattle, multiple similarities in the PK of flunixin regardless of method of administration have been observed (6, 15, 16, 29). This relationship has been identified to an extent in dairy goats (17), and recently in meat goats after transdermal administration (27). In dairy cattle, differences have been noted with respect to bioavailability, terminal half-life, and milk residue detection between different methods of injectable administration (29). Prolonged terminal half-lives are also associated with IM administration of FM to dairy cattle and goats (28, 30). The results of our study suggest similar terminal elimination half-lives of flunixin and 5-OH when flunixin is administered SC to lactating goats.

Due to a limited number of approved drugs available for treating goats in many countries, different classes of drugs licensed for sheep or cattle are extensively used in goats, in an extra-label fashion, without optimization of dosing regimens, determination of PK and pharmacodynamic (PD) properties, and estimated withdrawal intervals. It is generally acknowledged that plasma disposition and metabolism of anthelmintic drugs can be different between sheep and goats (31, 32). Previously reported PK parameters for sheep administered FM IV also differ from results from our study. Intravenous administration of FM to sheep yielded *T*_1/2λ*z*_ ranging from 2.5 to 3.8 h (33, 34), which were longer than those determined from our study's non-lactating goats, which ranged from 2.17 to 3.13 h. However, these *T*_1/2λ*z*_ for sheep administered FM IV were shorter than what we determined for lactating goats in our study (range of 4.35–4.56 h after IV administration).

Multiple age and reproductive status groups were incorporated into this study as the currently reported literature only demonstrated the PK of FLU in adult goats. In cattle administered FM, age was noted to alter PK parameters, primarily clearance being slower in calves at 2 months of age than at 8 months of age (26). A similar relationship was observed in the CL and CL/*F* in the group of young (nulliparous) does in this study (182 and 199 ml/kg/h) when compared to the older lactating does (265 and 365 ml/kg/h), but not when compared to the older pregnant non-lactating does (114 and 127 ml/kg/h). Specifically, when compared to the older pregnant non-lactating does, the younger nulliparous non-lactating does in our study demonstrated statistically significant differences, regardless of IV or SC route of administration, with CL and CL/*F P* values of 0.0019 and 0.0071, respectively. A higher CL and CL/*F* was noted in lactating does when compared to non-lactating does; this is a similar finding to flunixin clearance in lactating cattle (35). The extraction ratios appeared to vary between groups with NL pregnant does having a lower extraction ratio (1.44) than the younger nulliparous does or lactating does (2.08 and 3.55, respectively).

Part of this study's objective was to compare plasma concentrations determined by UPLC-MS/MS with concentrations quantified using commercial ELISA kit designed for bovine plasma. As reported in Table 3, no statistically significant differences were noted in concentrations determined by UPLC-MS/MS or ELISA from the goats within our study. This suggests that ELISA testing may be another useful approach for the determination of flunixin plasma concentration in goats, similar to what is reported for cattle (21). Future studies need to evaluate this diagnostic approach for caprine milk to determine if ELISA testing demonstrates similar results to plasma in this matrix. Additional testing modalities for flunixin in milk include lateral flow testing as determined for bovine milk, as well as gas chromatography coupled with mass spectrometry, which has also been identified as a suitable method for monitoring flunixin residues in goat milk (36–38).

In this study, SC FM injection sites were observed for swelling, heat, or pain. None of the goats administered FM SC in this study had adverse effects with respect to injection site reactions. For does in trial 3 (single dose, lactating does), they had an observed injection site score of 0 (no reaction) and 4 does had an injection site score of 1 (minimal reaction), with none of the scores of 1 lasting for longer than 48 h. All of the injection site scores resolved to 0 within 72 h. With this result, it appears that single SC doses of FM have minimal injection site reactions, whereas multiple SC doses would require monitoring by a producer or veterinarian. The lack of significant injection site reactions in these goats could be due to relative small drug volumes, location of injection, or undetermined factors. These results are promising since animal producers and handlers could administer the bovine IV formulation SC to goats for the treatment of pyrexia, provided the provisions of AMDUCA are met. Given that the Food Safety and Inspection Service (FSIS) has identified FM as an agent of high regulatory concern, any efforts to reduce the potential for residue violations would be beneficial for both producers and consumers of goat dairy products.

The methods for establishing withdrawal intervals presented in this manuscript provide an alternative method for estimating withdrawal intervals following extra-label drug use. In cattle, flunixin is one of the most common violative residues (4). Harmonization by the European Union allows for determination of milk residues by three methods: time to safe concentration, safe concentration based on linear regression, and safe concentration per milking (39). The data from our study do not represent those generated from a good laboratory practice study, nor do they meet the study design requirements of EMA or FDA for the drug approval process, but the regulatory statistical method provides a framework for estimating a conservative withdrawal interval following extra-label drug use. Part of the approach to our estimating the withdrawal intervals was to use a Monte Carlo simulation method to generate triplicate values for each milk sample as required by the FDA (40). By using the intra- and inter-assay coefficients of variation for the UPLC-MS/MS analytical method for milk, milk concentrations were simulated to fulfill the requirement of having triplicate values for each milk sample. The estimated milk withdrawal intervals were relatively close to the FDA-approved withdrawal time for dairy cattle when FM is administered. FM, as labeled for IV administration to dairy cattle in the United States, has a 36-h label milk withdrawal time when administered according to label directions. The current tolerance for 5-OH, the marker residue for flunixin in bovine milk in the United States, is 2 parts per billion (4). Similarly, the European maximum residue limit (MRL) is 4 parts per billion (41).

When the IV product is administered SC or intramuscularly to cattle, residues can be detected in the milk after 36 h, which is the withdrawal time for when the medication is administered IV, as indicated per label (29, 38). Our analysis suggests that using the EMA method estimated milk withdrawal intervals after SD IV, SD SC, and MD SC at 1.1 mg/kg dosing would be 36, 48, and 60 h, respectively. Similarly, using the FDA method, appropriate milk withdrawal intervals after SD IV, SD SC, MD IV, and MD SC at 1.1 mg/kg dosing would be 36, 36, 36, and 48 h, respectively. The estimated milk withdrawal intervals between the EMA and FDA methods were generally very similar. When rounding to the next milking interval assuming a 12-h milking interval, the estimated milk withdrawal intervals between the EMA and FDA methods were either the same (for SD IV) or different by only one milking interval (for SD SC and MD SC). Many studies report suggested milk withdrawal intervals based on a time concentration profile when milk residues are no longer detected. The challenge with these withdrawal interval suggestions are that they are based on a very small number of healthy study subjects. The advantage of our approach used in this study is that the FDA method reports when, with 95% confidence, 99% of animals will be free of residues (95% of animals for the EMA method). This approach to estimating a withdrawal interval recommendation following extra-label drug use is beneficial to clinicians since a greatly extended withdraw interval needs to be provided to assure food safety.

Additional research is necessary for the PD of flunixin in goats, and what effect, if any, SC administration has on PD. In a study evaluating transdermal FM in goats, the 80% inhibitory concentration was 0.28 μg/ml, but was only achieved with IV administration (27). In a study evaluating prostaglandin synthesis following IV, IM, and oral administration of FM to dairy goats, a decrease of prostaglandin plasma concentration was noted regardless of the method of administration of FM (17). Another study identified an inhibition of the generation of thromboxane B2 in goat blood when flunixin was added (42). One study noted higher pain scores in a control group compared to an epidural morphine-treated group in goats undergoing abdominal surgery; in that study, the control group pain scores were lowered by post-operative flunixin administration (43). In cattle, flunixin has been demonstrated to decrease inflammation, as well as pain from castration, disbudding, and lameness; however, limited PD data exist for flunixin in goats.

This study had several limitations. While the study population was distributed into multiple ages as well as lactation statuses and stages to simulate a commercial dairy setting, primarily only five breeds of dairy goat were represented in our study population. This study also used a relatively small number of healthy goats, and while this is commonly done for PK studies, recent research has questioned this practice for determining withdrawal intervals for flunixin in large diverse populations of cattle (44, 45). Since the design of this study does not meet the regulatory requirements of EMA or FDA, the estimated milk withdrawal intervals from this study can only serve as a preliminary recommendation. Additional studies that meet the EMA or FDA study requirements are needed in order to make a more conclusive scientifically based recommendation. The PK parameters in this study were from healthy goats, and caution would have to be used when applying estimated withdrawal intervals to milk samples from sick goats as disease could influence milk withdraw periods as well as milk production. The comparison of PK parameters based on age did not allow the same animals to serve as their own control. Milk elimination is prolonged in cattle with mastitis vs. healthy cattle (46). A prolonged elimination could pose a food safety risk as it would increase the potential for a violative drug residue. In other species, large variations in body size could necessitate dose adjustments based on an allometric function of body weight (47). This study also assumes that dairy goats are similar to cattle in that 5-OH is the major metabolite/marker residue, as 5-OH is the predominant residue with 4-OH flunixin and 2-OH methylflunixin being minor residues (48). Future studies could also incorporate non-linear mixed effects modeling and/or physiologically based pharmacokinetic (PBPK) modeling, as these techniques can account for variability for optimization of dosing schedules and serve as an additional tool for withdrawal time determination (49, 50).

## Conclusions

The PK profile of SC FM described in this study including the similar elimination half-lives supports clinical evaluation of the SC administration of this drug for management of pain and inflammation in goats. ELISA testing of goat plasma for FLU concentrations may provide similar results to UPLC-MS/MS. Goat milk concentrations of FLU and 5-OH appear to be similar for both IV and SC routes of administration. After SC administration, a milk withdrawal interval of 36–60 h should be considered depending on the dose and dosing frequency. For dairy goats, SC FM may prove to be a clinically useful method of administration on the farm where allowed by appropriate extra-label drug use provisions.

## Data Availability Statement

All datasets generated for this study are included in the article/supplementary material.

## Ethics Statement

The animal study was reviewed and approved by Institutional Animal Care and Use Committee, University of California, Davis.

## Author Contributions

JS was involved in study concept and design, data collection, flunixin analysis, data analysis, and manuscript preparation. JA participated in study concept and design, data collection, animal procurement, and manuscript preparation. ZL participated in milk withdrawal interval time modeling and calculation, as well as manuscript preparation. JR contributed to data collection and manuscript preparation. TM was involved in data collection and manuscript preparation. EL participated in data collection, animal procurement, and manuscript preparation. JC contributed to animal procurement and data collection. WS contributed to ELISA analysis and manuscript preparation. LT originated the study design and concept for using Monte Carlo simulation method for generating milk data points for FDA and EMA regulatory methods, sample collection, data analysis, and manuscript preparation. All authors have read and approved the final manuscript.

## Conflict of Interest

The authors declare that the research was conducted in the absence of any commercial or financial relationships that could be construed as a potential conflict of interest.

## Abbreviations

*C*_max_: Maximum concentration (observed)
CV: Coefficient of variation
CVMP: Committee for Veterinary Products for Medical Use
ELISA: Enzyme-linked immunosorbent assay
EMA: European Medicines Agency
FDA: Food and Drug Administration (USA)
FLU: Flunixin
FM: Flunixin meglumine
IV: Intravenous
LOD: Limit of detection
LOQ: Limit of quantification
MD: Multiple dose
LC-MS/MS: Liquid chromatography mass spectrometry
NL: Non-lactating
NSAIDS: Non-steroidal anti-inflammatory drugs
PD: Pharmacodynamic(s)
PK: Pharmacokinetic(s)
SC: Subcutaneous
SD: Single dose
*T*_1/2λ*z*_: Terminal elimination half-life
*T*_max_: Time to maximum concentration (observed)
UPLC: Ultra-performance liquid chromatography
USA: United States of America
USDA: United States Department of Agriculture
5-OH: 5-Hydroxy flunixin.
